# How trust in coworkers fosters knowledge sharing in virtual teams? A multilevel moderated mediation model of psychological safety, team virtuality, and self-efficacy

**DOI:** 10.3389/fpsyg.2022.899142

**Published:** 2022-09-02

**Authors:** Qi Hao, Bin Zhang, Yijun Shi, Qizhong Yang

**Affiliations:** ^1^The School of Information Resource Management, Renmin University of China, Beijing, China; ^2^College of Foreign Languages and Cultures, Beijing Wuzi University, Beijing, China; ^3^The School of Arts and Sciences, University of Rochester, New York, United States

**Keywords:** knowledge sharing, virtual team, trust in coworkers, psychological safety, self-efficacy, team virtuality

## Abstract

Examining the influence of trust in fostering knowledge sharing behavior (KSB) in virtual teams is of great research value in the current complex, dynamic, and competitive era of a knowledge economy. This study investigated the relationship between trust in coworkers (TC) and KSB. Based on social information processing theory and social cognitive theory, we developed a multilevel moderated mediation model where the team members’ psychological safety (PS) was considered a mediator, while team virtuality (TV) and knowledge sharing self-efficacy (KSSE) acted as team and individual-level moderators, respectively. On surveying 282 individuals in 37 virtual teams of three Chinese internet companies, we found that TC positively affected team members’ KSB and this relationship was fully mediated by team members’ PS. Our findings also demonstrated that the effect of TC on KSB depended on the degree of TV and employees’ KSSE. Specifically, when TV and KSSE were higher, the TC–PS and PS–KSB relationship and the mediating effects of PS in the TC–PS–KSB relationship were all stronger. Our study extends the trust-KSB literature by identifying the psychological mechanism and boundary conditions in the TC-KSB relationship. Moreover, our findings also offer valuable managerial implications for virtual team managers on facilitating team members’ PS and KSB.

## Introduction

The rapid proliferation of computer-mediated communication (Peñarroja et al., 2015), the complex and hypercompetitive nature of the business environment (Frazier et al., 2017), and the threat and surge in the Coronavirus disease (COVID-19) pandemic (Whillans et al., 2021) make the adoption of a new organizational form—virtual team becoming more prevalent and urgent. A virtual team refers to “a group of individuals who are geographically dispersed, have limited face-to-face contact, and work interdependently through electronic mediums to achieve a shared objective” (Hao et al., 2019b, p: 43). Literature indicates the benefits of using virtual teams. Hiring knowledge workers in geographically dispersed regions, increasing global workday from 8 to 24 h, slashing travel costs, and enabling knowledge exchanging across organizational boundaries (Pangil and Chan, 2014; Hao et al., 2019b), can promote competitive advantage and achieve long-term success among organizations (Dulebohn and Hoch, 2017). A 2016 survey reported that over 80% of the respondents contended that a virtual team is vital to accomplish their work (RW3 CultureWizard, 2016). In addition, more recent data showed that about 50% of US employees have been working in virtual teams since March 2020, owing to COVID-19 (Whillans et al., 2021). However, the popularity and promise of virtual teams raises an important question: what makes a virtual team more efficient and vibrant? Previous work argued that team members’ knowledge sharing behavior (KSB) was a salient factor affecting virtual team effectiveness (Pangil and Chan, 2014). Similarly, some scholars argued that the value of a virtual team is limited without abundant knowledge (Fang and Chiu, 2010). Furthermore, a study showed that nearly “50 percent of virtual teams would fail to meet either strategic or operational objectives due to the inability to” effectively manage knowledge exchange, transfer, and sharing among distributed workforce (Zakaria et al., 2004, p: 17). Thus, recognizing the factors facilitating knowledge sharing among virtual team members has significant research value.

Multiple factors affect KSB within a virtual team or other virtual environments have been identified in the existing literature, ranging from individual perspectives (e.g., personality, core self-evaluations, motivation, trust, and self-efficacy) to situational forces, such as work environment, organizational support, leadership, team characteristic, and job design (Ardichvili et al., 2003; Wasko and Faraj, 2005; Chen and Hung, 2010; Pee and Lee, 2015; Hao et al., 2019b). Among these, trust has been studied extensively (e.g., Jarvenpaa and Leidner, 1999; Ridings et al., 2002; Fang and Chiu, 2010; Pangil and Chan, 2014). Scholars agreed that building trust was significant for promoting KSB in virtual environments (Hsu et al., 2007). Despite this progress, a systemic review of the trust–KSB literature unfolds that most previous scholars have emphasized the effects of vertical trust relations (i.e., trust in leaders or trust in organizations) on KSB, relatively little attention has been assigned to horizontal trust relationships, such as trust in coworkers (TC; Lin, 2007; Tan and Lim, 2009; Fang and Chiu, 2010). This raises concerns as the prevalence of virtual teams and the tasks are becoming increasingly interdependent. Previous study argued that information from surrounding others (e.g., colleagues) can offer critical social cues for employees shaping their views, attitudes, and behaviors (Lau and Liden, 2008). Given that trustworthy coworkers create open, comfortable, and unambiguous social context for employees, social information processing theory (SIPT; Salancik and Pfeffer, 1978) can serve as a theoretical perspective to help explore the association between TC and KSB. The tenet of SIPT is that individuals usually use the information obtained from their immediate social contexts to construct and interpret realities, especially when the condition is ambiguous and uncertain, such as virtual environment (Salancik and Pfeffer, 1978; Lin et al., 2022). Thus, drawing on SIPT, we expect that TC may play an important role in facilitating virtual team members’ KSB. More specifically, we propose two research questions: (a) what is the intervening mechanism underlying the relationship between TC and KSB in virtual teams? and (b) are there some boundary conditions that may alter how TC affects KSB?

To address the first question, we examined the psychological mechanism through which TC affects employees’ KSB. According to SIPT, the social information emitted by coworkers helps to shape an employee’s perception on work environments. Individuals who surrounded by trustworthy coworkers are likely to have fewer worries about the possible undesirable consequences of their actions (Zhang et al., 2010), leading to high levels of psychological safety (PS). PS is defined as “feeling able to show and employ one’s self without fear of negative consequences to self-image, status, or career” (Kahn, 1990, p: 708). It is considered a critical psychological mechanism, which connects trust and outcomes (Roussin, 2008; Zhang et al., 2010; Basit, 2017). In addition, previous studies showed that PS is significantly associated with voice behaviors (Frazier and Bowler, 2015), information sharing (Bunderson and Boumgarden, 2010), and knowledge hiding (Men et al., 2020). Thus, we expect TC to have implications on virtual team members’ PS, which, in turn, facilitates knowledge sharing among team members.

To address the second research question, we extend the presumed TC–PS–KSB model by identifying two important boundary conditions at different levels: team virtuality (TV) at the team level and knowledge sharing self-efficacy (KSSE) at the individual level. As highlighted by SIPT, employees’ attitudes and behaviors can be influenced by social cues from both significant individuals (e.g., colleagues) and workplace environmental characteristics (Lin et al., 2022). Thus, different working environments could offer some important information for employees to process (Salancik and Pfeffer, 1978). In other words, the effect of TC on PS and subsequent KSB could fluctuate under different working conditions. The most important environmental characteristic of virtual teams is TV which refers to “the degree to which members use technology to interact across geographic, organizational, or other boundaries” (Bierly et al., 2009, p: 551). Based on SIPT, different degrees of TV may send different signals which help virtual team employees perform specific work-related behaviors. More specifically, we argue that the social cues emitted by highly virtuality working context, such as unreliable and complex telecommunication technologies, low likelihood of informal team member communication, and highly risky perception of collaboration (Bierly et al., 2009; Ganesh and Gupta, 2010; Breuer et al., 2016), may shift virtual team members’ attention to the significant information of their surrounding others (e.g., coworkers) that provides a clear path to shape their cooperative attitudes and behaviors. Thus, in the presence of highly virtuality working context, virtual team members are more inclined to process the information from trustworthy coworkers to foster higher degrees of PS and subsequent KSB.

From an individual perspective, the social cognitive theory suggests that human behavior is influenced by a triadic and dynamic system including individual factors, behaviors, and interpersonal network (Bandura, 1982; Chiu et al., 2006). One core factor of the theory is self-efficacy referring to “a judgment of one’s ability to organize and execute given types of performances” (Chiu et al., 2006, p: 1873). In the knowledge sharing domain, previous studies indicated that an employee’s belief in their capability to perform KSB successfully (i.e., KSSE) is a pivotal predictor for knowledge sharing in virtual environments (Hsu et al., 2007; Hao et al., 2019b). KSSE can provide a “can do” attitude, which may intensify the positive effect of the “reason to” factor, such as TC and PS in this study, on KSB (Hao et al., 2019a). Thus, consistent with previous studies (Hao et al., 2019a,b), we investigated the individual-level boundary conditions of the TC–PS–KSB relationship by testing the moderating role of KSSE.

**Figure 1:**
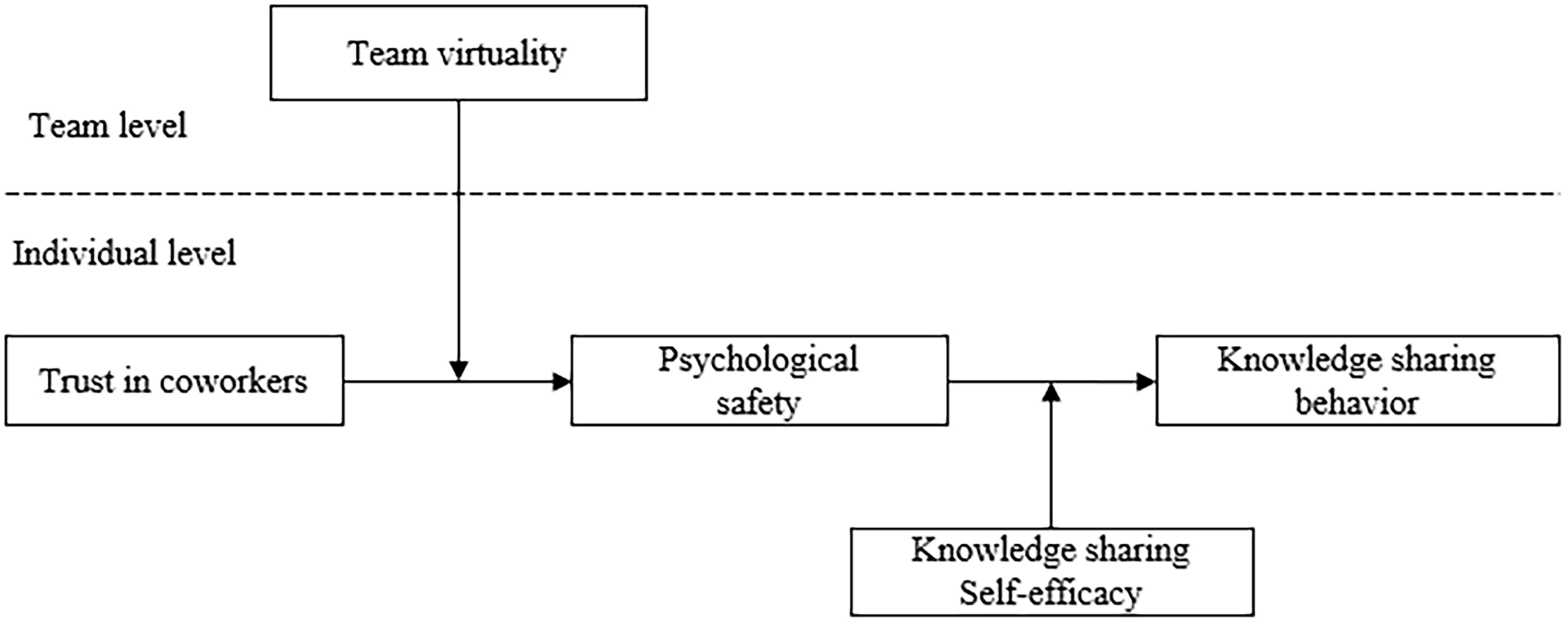
Research model.

Collectively speaking, drawing upon SIPT and social cognitive theory, we developed a multilevel moderated mediation model to uncover the complex and dynamic nature of the TC–KSB relationship in virtual teams. PS mediates the relationship between TC and KSB. Moreover, TV and KSSE act as contingencies to alter the TC–PS–KSB link at the team land individual levels, respectively (see Figure 1). Our theoretical viewpoint and empirical results provide multiple contributions to the existing literature. First, based on SIPT, our study extends the trust-KSB literature by examining the overlooked horizontal mutual trust in virtual teams (i.e., TC). Second, our study unfolds the complex and dynamic nature of the TC–KSB relationship by introducing PS as a mediator. KSB could be a desirable consequence of team members’ progressively enhanced PS induced by the processing of social cues emitted from trustworthy coworkers. Third, our study identifies the boundary conditions that can enhance the effects of TC and PS at different research levels (i.e., team level and individual level) base on SIPT and social cognitive theory, respectively.

## Theory and hypotheses

### KSB in virtual teams

Knowledge is considered a vital resource that underpins an organization’s competitive advantage and long-term success (Pereira and Mohiya, 2021). In this regard, managers need to manage knowledge diligently for expanding organizational knowledge boundaries. During the past decades, the rapid progress of the information technology and the growing popularity of online instant messengers allowed organizations to establish virtual teams. These teams helped solve the unevenly distributed knowledge problems by combining the geographically dispersed knowledge workers (Cohen and Bailey, 1997; Pangil and Chan, 2014). Virtual teams can offer a flexible and responsive platform for global team members with different competencies to exchange and share their knowledge across time and space constraints and organizational boundaries (Jarvenpaa and Leidner, 1999). Scholars argue that setting up a virtual team can hardly guarantee smooth knowledge sharing among employees and significant organizational knowledge accretion (Davidavičienė et al., 2020). However, there are merits to virtual teams. They are easily accessible, flexible, responsive, and cost-effective (Jarvenpaa and Leidner, 1999; Pangil and Chan, 2014). However, KSB requires individuals to convert their own expertise and unique knowledge “into a form that can be readily understood, absorbed, and employed by others” (Hao et al., 2019b, p: 43). This process may cost knowledge providers’ precious time and energy, reduce their competitiveness in an organization, and eventually land them in a risky situation (Lee et al., 2018). Thus, KSB among employees may not be possible in the absence of solid trust between knowledge providers and recipients (Lin, 2007). In contrast, the dysfunctions of virtual teams such as low individual commitment, social loafing, loss of non-verbal cues, cultural estrangements, changes of members, and complicated technologies (Jarvenpaa and Leidner, 1999; Pangil and Chan, 2014; Davidavičienė et al., 2020) make trust more difficult to build in virtual teams than in traditional ones. In addition, a previous study indicated that fresh virtual team members usually need a long time to fully trust, cooperate, and share with others (Kanawattanachai and Yoo, 2007). Therefore, trust has been highlighted as an important factor in facilitating KSB under virtual contexts (e.g., Jarvenpaa and Leidner, 1999; Hsu et al., 2007; Fang and Chiu, 2010).

### TC and KSB

Trust is defined as “an implicit set of beliefs that the other party will refrain from opportunistic behavior and will not take advantage of the situation” (Ridings et al., 2002, p: 275). It has been widely studied in research fields, such as e-marketing, intellectual capital management, and knowledge management (Zhang et al., 2010). Previous studies showed that trust significantly affects online transactions, organizational value creation, job satisfaction, organizational commitment, and knowledge sharing (Chiu et al., 2006; Zeinabadi and Salehi, 2011). Despite the abundant literature and clear consensus on the critical role of trust, most of these studies have focused on trust in vertical referents, such as supervisor, manager, and organization (e.g., Deluga, 1994; Breuer et al., 2016), and seldom examined the horizontal referents, such as coworkers (Tan and Lim, 2009).

The fundamental premise of SIPT is that “individuals, as adaptive organisms, adapt attitudes, behavior, and beliefs to their social context and to the reality of their own past and present behavior and situation” (Salancik and Pfeffer, 1978, p: 226). In the workplace, employees are likely to seek and use information from observations of their colleagues and environmental characteristics to shape and comprehend their reality and subsequently to perform specific work-related behaviors (Salancik and Pfeffer, 1978; Lin et al., 2022). Drawing upon on SIPT, we argue that examining TC in virtual teams is important and necessary. First, the increasing prevalence of flat organizational forms requires managers to emphasize the quality and effectiveness of horizontal interactions between coworkers in an organization (Singh et al., 2019). These social cues are important materials for employees shaping work-related attitudes and behaviors. Second, the interdependent nature of virtual team tasks facilitates sharing of common responsibilities among members who are under team-oriented reward and penalty systems (Lau and Liden, 2008). A virtual team member may process these signals and find that whether they can be rewarded or punished is dependent on the actions and efforts of other members. Thus, TC is especially crucial for their efforts and work-related behaviors (Lau and Liden, 2008). Third, SPIT suggests that when employees get social information from individuals that are similar to them, they may regard these social cues more salient (Tan and Lim, 2009). The interactions between coworkers are characterized by little power imbalance, which exhibits horizontal dynamics that are absent in vertical relationships between subordinates and authoritative supervisors (Tan and Lim, 2009). Thus, virtual team members’ attitudes and behaviors are more likely to influenced by the social information comes from coworkers whom are considered as similar to themselves. Following these arguments, this study examined the effect of TC on virtual team members’ work-related behaviors (e.g., KSB) through the theoretical lens of SIPT.

Employees’ TC stems from their interaction qualities with colleagues (Lin, 2007). If employees perceive a colleague to possess trustworthy attributes such as ability, benevolence, or integrity, they are likely to trust them (Mayer et al., 1995; Tan and Lim, 2009). Based on SIPT, the reason why TC can significantly predict virtual team members’ KSB is twofold. First, a salient barrier to share knowledge is individuals’ natural reluctance to participate in risky activities because sharing personal knowledge is considered a form of sharing power with colleagues (Ardichvili et al., 2003; Lin, 2007). When people are surrounded by trustworthy coworkers, they may get the social cues that their actions can lead to favorable outcomes (Davidavičienė et al., 2020). After processing these social cues, employees will construct a safety reality which reduces their uncertainty perception, wipes out unwelcomed and opportunistic behaviors, and promotes risk-taking (Hsu et al., 2007; Lin, 2007). Similarly, a previous study by Jarvenpaa and Leidner (1999) indicated that TC could effectively prevent virtual team members’ geographic distance from becoming psychological distance, enhancing their engagement in collaborative behaviors, such as KSB. Second, according to SIPT, individuals’ work-related behavior cannot occur comes out of thin air, and can be influenced by certain circumstances. KSB is considered a type of additional behavior, which is “not formally prescribed by organizations, difficult to measure, and problematic to formally appraise” (Hao et al., 2019a, p: 2768). Previous studies showed that TC acts as a subjective norm to substitute formal or legal systems guaranteeing employees perform expected activities (Mayer et al., 1995; Chiu et al., 2006). This social information determines the acceptability and appropriateness of performing a given behavior through sensemaking process. Thus, TC is particularly critical in facilitating virtual team members’ volitional and discretionary behaviors (e.g., KSB). Consistent with the above theories, we propose:

*H1*: TC has a positive effect on virtual team members’ KSB.

### The mediating of PS

PS describes individuals’ intrapsychic states associated with interpersonal experience, which reflects employees’ perceptions and taken-for-granted beliefs about the risk level of their surrounding interpersonal environment (Edmondson et al., 2004; Zhang et al., 2010). When employees possess a high level of PS, they are likely to believe that their behaviors and activities will not produce undesirable consequences. In the process, they are encouraged to ask questions, propose new ideas, solicit feedbacks, express themselves, and share work-related skills (Edmondson et al., 2004; Zhang et al., 2010; Frazier et al., 2017). Due to the substantial benefits of PS, creating this positive state is a central issue in psychological and organizational research on PS. Previous studies revealed that various factors could predict PS, such as personality traits, leader behaviors, interpersonal trust, organizational support, and work design (Kahn, 1990; Frazier et al., 2017). Among these, this study will focus on how TC affects virtual team members’ PS.

TC is a critical element for determining team members’ attitudes and behaviors, especially in virtual teams. SIPT suggests that social context can shift an employee’s attention on certain information and make this information more salient (Salancik and Pfeffer, 1978). Virtual team members usually lack of face-to-face communications and visual cues. They share little prior working together experience and have different cultures and languages (Jarvenpaa and Leidner, 1999; Ridings et al., 2002). These social cues may help focus an individual’s attention on other information sources, for example other virtual team members, and provides expectations about individual attitudes and behaviors. Following this logic, if virtual team members do not trust each other, they may fall into a threatening interpersonal context, significantly reducing their PS. In contrast, when people are in a trusting working environment, they are likely to attempt more activities without worrying about consequences because the social cues they obtained help them construct the belief that any feedback from coworkers would be kind and constructive (Kahn, 1990; Zhang et al., 2010). In this regard, TC can effectively release individuals’ burdens about possible negative outcomes of their work-related behaviors, which elevates their PS level (Zhang et al., 2010). Consistent with these arguments, we assume a positive association between TC and PS.

Previous studies indicated that an important barrier to not sharing knowledge in virtual environments is that knowledge providers fear losing face or providing imperfect knowledge (Ardichvili et al., 2003). Since PS can minimize the potential undesirable ramifications of voicing ideas or making mistakes (Frazier et al., 2017), employees with a high level of PS may be more willing to raise their opinions and share work-related knowledge and skills with others. In addition, high PS offers people the perception of “less threatened by exposure to the judgment of the recipient” (Men et al., 2020, p: 464), which can promote employees’ frequent interactions with other coworkers. These frequent and smooth communications among employees can foster a favorable sharing climate, thus facilitating KSB (Siemsen et al., 2009). Therefore, we expect that PS can positively affect employees’ KSB.

Integrating these arguments suggests that TC can serve as a source of social context facilitating employees’ rise of PS state where they may feel their coworkers are kind. Any criticism from coworkers would be constructive and well-meant. Consequently, they are willing to express themselves, communicate with others, and proactively share their knowledge and skills. Therefore, we propose:

*H2*: PS mediates the relationship between TC and KSB, such that TC first affects PS, which in turn affects KSB.

### The moderating role of TV

The concept of TV stems from the increasingly entrenched organizational form of virtual team, which is adopted to depict the extent to which a team is virtual (Bierly et al., 2009). Several companies have established multinational subsidiaries and outsourcing has become more prevalent; hence, the need for deploying virtual teams has increased sharply (Ganesh and Gupta, 2010). However, the widespread use of information and communication technology in work teams makes it hard to determine if a team is purely virtual or traditional face-to-face (Ganesh and Gupta, 2010). Thus, it is necessary to study TV as an inherent team attribute rather than just making a binary distinction, that is, virtual and face-to-face teams. Previous studies adopted different criteria to measure TV, and most authors conceptualize TV as a multidimensional variable (Foster et al., 2015). For example, Chudoba et al. (2005) measure TV using three dimensions: team distribution, workplace mobility, and variety of practices. Kirkman and Mathieu (2005) assessed a team’s TV according to three criteria: (a) the extent to which team members rely on virtual tools, (b) the extent to which the online data and information are valuable for a team, and (c) the extent to which synchronous interactions occur.

In addition to the research of TV measuring, previous research highlighted that the degree of the virtuality of a team affects participative behaviors, such as information sharing and cooperation (Bierly et al., 2009; Mesmer-Magnus et al., 2011). These studies viewed high TV as a double-edged sword in such relationships: on the one hand, highly virtual environments may reduce the suppression coming from social norms and group pressures, conveniently access to team members using instant messengers, easily record the communication data, and allow sufficient time to consider and digest other members’ shared knowledge; on the other hand, high levels of TV may negatively affect collaborative behaviors because of its disadvantages, such as little non-verbal cues, difficult in coordinating, potentially disjointed communication, and high technical threshold (Mesmer-Magnus et al., 2011; Foster et al., 2015).

This study assumes that TC and TV can influence virtual team members’ PS using an interactionist approach. According to SIPT, employees may construct meaning of reality and develop certain attitudes and behaviors based on social information both from significant coworkers and working contexts (Lin et al., 2022). Specifically, SIPT suggests that when the social context is “uncertain, ambiguous, and complex, team members will more likely rely on social cues to from their individual beliefs and attitudes” (Lau and Liden, 2008, p: 1132). In other words, the characteristics of work conditions can make the social cues from individuals more significant for processing (Lin et al., 2022). In our case, when employees working in high TV contexts, they are highly dependent on electronic tools, reducing social cues and controls and producing conflict problems, such as delayed responses and neglection of important information, thereby increasing their perceptions of collaboration risks (Jarvenpaa and Leidner, 1999). These team members may face role ambiguity and workplace misattributions (Breuer et al., 2016). In such chaotic context, the trustworthy colleagues can offer strong social information that employees are unrestrained being themselves and more likely to be involved in interpersonal communications (Men et al., 2020). In contrast, low TV teams (e.g., face-to-face team) represents social information that team members have more opportunities to interact with each other in informal ways, such as accidental conversations at coffee machines (Jarvenpaa and Leidner, 1999). These informal interactions may make the working circumstance clearer and more transparent, which diminishes TC’s role in influencing PS. Taken together, through the above social information processing, people in highly virtual contexts may have stronger risk perceptions than those in a low TV level, which implies the need for increased importance of TC. This is reflected in a stronger association between TC and PS. Therefore, we propose:

*H3*: TV can moderate the relationship between TC and PS; for teams with higher level of TV, TC will more positively affect PS.

A study by Edwards and Lambert (2007) suggested that if a variable plays a moderating role in the relationship between the independent variable and mediator or that between the mediator and dependent variable, this variable can affect the whole mediated model. Thus, in this study, we proposed a moderated mediation model by combining the presumed mediating model of TC–PS–KSB with the moderating role of TV in the TC-PS relationship. Specifically, employees in higher TV environments are more likely to generate collaborative risk making TC stronger in elevating PS. In this situation, these employees may perform more KSB because of the increasing level of PS. In other words, PS plays a more important role in connecting TC and KSB in higher TV contexts (compared with lower TV contexts). Thus, we propose:

*H4*: TV can moderate the mediation relationship of TC with KSB through PS; for teams with higher level of TV, the mediating effect of PS will be stronger.

### The moderating role of KSSE

When robust trust relationships have been established among virtual team members, individuals may relax their vigilance and generate safe and worry-free feelings in their minds, which provides them with an important reason to exhibit KSB. However, having reasons for sharing is not a sufficient reason for KSB. Instead, a knowledge provider’s perception of possessing capabilities to complete KSB, known as a “can do” attitude, is considered a pivotal amplifier in the sharing process (Hsu et al., 2007; Hao et al., 2019a). Therefore, we adopted KSSE as a typical form of this “can do” attitude.

According to social cognitive theory, self-efficacy is idiosyncratic construct that can affect the decision making of what behaviors to undertake, the amount of effort to overcome faced obstacles, and mastered perception of a behavior (Bandura, 1982; Hsu et al., 2007). People with higher self-efficacy are inclined to achieve related behavior better than those with lower self-efficacy. More recently, some knowledge management scholars have applied self-efficacy in the knowledge sharing field, creating a new concept—KSSE to “validate the effect of personal efficacy belief in knowledge sharing” (Hsu et al., 2007, p: 155). They argued that people with high KSSE usually hold the beliefs that they possess capabilities, such as authoring useful and accurate knowledge content, adding related context to knowledge to transform it into comprehensible forms, and clearly communicating knowledge to recipients with or across work teams, to successfully implement KSB (Hsu et al., 2007). Thus, KSSE is considered a critical self-motivational factor for KSB in previous studies (e.g., Kankanhalli et al., 2005; Hao et al., 2019b).

This study employed an interactionist perspective to propose that KSSE and PS may have a combined effect on virtual team members’ KSB. Employees with higher PS levels usually believe that their surrounding contexts are safe and comfortable; thus, they are willing to communicate and share expertise with others (Men et al., 2020). However, social cognitive theory suggests that individuals’ positive expectations of a certain behavior will be fruitless if they doubt their ability to successfully carry out this behavior (Hsu et al., 2007). In this study, according social cognitive theory, we argue that employees only have the willingness to share knowledge is not enough for carrying it out, they must also have the confidence to accomplish it. Thus, in instances where these employees experience a lack of useful and accurate knowledge for organizations or a dearth of abilities to convert their knowledge into an accessible form (i.e., lack of KSSE), they may refrain from exhibiting KSB. They may consider their contribution is useless for organizational development, which in turn produces a detrimental effect on the PS-KSB relationship. Conversely, if these employees have a higher level of KSSE, the positive effects of PS on KSB will be amplified because they have the psychological motivation to perform more KSB and believe in their capabilities to execute KSB successfully. In sum, we propose:

*H5*: KSSE can moderate the relationship between PS and KSB; for individuals with higher level of KSSE, PS will more positively affect KSB.

According to the method of H4, assuming a moderation effect of KSSE in the PS-KSB relationship, it is also likely that KSSE can conditionally influence the indirect relationship between TC and KSB, thereby typically demonstrating a moderate mediation model among these variables. Since we presumed a stronger (weaker) relationship between PS and KSB when employees have higher (lower) KSSE, we hypothesize:

*H6*: KSSE can moderate the mediation relationship of TC with KSB through PS, for individuals with higher level of KSSE, the mediating effect of PS will be stronger.

## Research methodology

### Sample and procedures

Data were collected from three internet enterprises in China using an internet survey. These three companies have multiple branches at home and abroad; moreover, most employees work in project teams or R&D teams with varying degrees of virtuality. In addition, the team members need to frequently exchange knowledge, thoughts, and skills to achieve their tasks because these companies are all knowledge-intensive. Thus, the sample is relevant for studying KSB in virtual teams. The survey was conducted with assistance from the coordinators from the companies in two waves with a time interval of 6 weeks to reduce the potential common method bias (CMB) and priming effect of cross-sectional research design, according to previous suggestions (Podsakoff et al., 2003).

In the first wave, we asked our coordinators to directly send e-mails to the potential individuals (*N* = 410). The sent e-mails comprised (a) an introduction about the survey and the confidentiality of the collected data, (b) a web link of the questionnaire, and (c) a unique code number which the participants were required to add at the end of the questionnaire to match the two-wave survey. In this phase, the participants were asked demographic information (i.e., age, sex, and education), team name, team size, and to rate TC, PS, TV, and KSSE levels. We collected 371 valid questionnaires in this phase. After 6 weeks, we conducted the second-wave survey. The coordinators sent 371 e-mails to the employees who participated in the first-wave survey to assess their KSB levels. Finally, we collected a dataset including two hierarchically nested levels: 282 employees (individual level) nested in 37 teams (team level), for a final response rate of 68.8%. The average age of the respondents was 29.4 years (SD = 5.22), and most of them were male (73.6%). A vast majority of the respondents were highly educated (95.4% had a bachelor’s degree or higher). The smallest and the biggest team had 5 and 14 members, respectively, and the mean size was 7.62 (SD = 2.31) members per team.

We also conducted an independent sample t-test to investigate whether significant differences were existed in study variables between employees who both participate in the two waves and those who do not. Results indicates that women are more likely to drop out (*t* = 3.31, *p* < 0.05) and no significant differences exist for the other main study variables.

### Measures

All measures were adopted from research published in leading journals. We made minor modifications to adapt them to our research background. Moreover, we employed the translation-back-translation procedure to translate the measures into Chinese. Unless otherwise specified, all constructs were measured on a five-point Likert-type scale, ranging from 1 (strongly disagree) to 5 (strongly agree). TC was measured using Lin (2007) 5-item scale. PS was measured using Liang et al. (2012) 5-item scale. TV was measured using Chudoba et al. (2005) 12-item scale. The scale comprises three dimensions of virtuality, namely team distribution (4 items), workplace mobility (5 items), and variety of practices (3 items). KSSE was measured using Lin et al. (2009) 3-item scale. KSB was measured using Lin et al. (2009) 3-item scale. The questionnaire items of all the scales are listed in Appendix A.

In line with Men et al. (2020), respondents’ demographic characteristics were controlled in this study. In addition, we also controlled team size because a previous study showed that larger team size might affect KSB within teams (Zhao et al., 2016).

## Results

### CMB

CMB may be a potential problem for our results because our data was self-reported from a single source (Podsakoff et al., 2003). Thus, we employed the single-factor test to address this problem. The result showed that no single factor could interpret more than 29.4% of the total variance, indicating CMB was not a major problem in the study.

### Measurement model

We tested the convergent and discriminant validity of the model. The results showed that the factor loadings were all above 0.70, the composite reliability (CRs) ranged from 0.84–0.94, the Average variance extracted (AVEs) ranged from 0.58–0.66, the Cronbach’s α ranged from 0.83–0.91, and the square root of each construct’s AVE was greater than all correlation coefficients of the construct and other constructs (see Tables 1, 2). In addition, we also computed the heterotrait-monotrait (HTMT) ratio for each pair of constructs based on the item correlations (Henseler et al., 2015). The results showed that all the values were below 0.85 (see Table 2). According to previous suggestions (Fornell and Larcker, 1981; Clark and Watson, 1995), our model’s convergent and discriminant validity were good.

**Table 1:** Convergent validity and reliability analysis.

Constructs	Number of items	Factors loading range	Composite reliability (CR)	Average variance extracted (AVE)	Cronbach’s α
TC	5	0.72 ~ 0.85	0.90	0.64	0.89
PS	5	0.71 ~ 0.87	0.90	0.65	0.91
TV	12	0.70 ~ 0.83	0.94	0.58	0.85
KSSE	3	0.77 ~ 0.80	0.85	0.62	0.83
KSB	3	0.78 ~ 0.84	0.84	0.66	0.87

*N*_I_ = 282. *N*_T_ = 37.

**Table 2:** Correlations and HTMT ratios of each pair of constructs.

Variables	Mean	SD	1	2	3	4	5
1. TC	3.77	0.61	(0.80)	0.31^**^	−0.10	0.08	0.27^**^
2. PS	3.73	0.64	**0.45**	(0.81)	−0.02	0.18^**^	0.38^**^
3. TV	3.59	1.01	**−0.17**	**−0.03**	(0.76)	0.04	0.12^*^
4. KSSE	3.88	0.70	**0.21**	**0.36**	**0.16**	(0.79)	0.23^**^
5. KSB	3.91	0.81	**0.40**	**0.58**	**0.26**	**0.38**	(0.81)

*N*_I_ = 282. *N*_T_ = 37. Square roots of AVE are displayed on the diagonal in parentheses. Values in bold are HTMT ratios of each pair of constructs.

* *p* < 0.05;

** *p* < 0.01.

### Hypotheses testing

Given the cross-hierarchical nature of our data, we employed Hierarchical Linear Modeling (HLM) version 8.0 to test our hypotheses. Prior to testing the hypotheses, we first validated the aggregation of individual-level measures of TV on the team level by computing the intra-class correlations (ICCs) and the multi-item within-team agreement (r_wg(J)_). The results showed that ICC1 and ICC2 was 0.23 and 0.59 (*F* = 2.48, *p* < 0.001), respectively, and r_wg(12)_ ranged from 0.71–0.96 with a mean value of 0.82, indicating that HLM is an appropriate analytic method.

The hypotheses testing results are presented in Table 3. The results of model 5 showed that TC had a significant effect on KSB (M5; *γ* = 0.22, *p* < 0.01). Thus, H1 was supported.

**Table 3:** Hierarchical regression results.

Intercept and variable		PS			KSB			
		M1	M2	M3	M4	M5	M6	M7
Intercept		3.73^**^	3.73^**^	3.74^**^	3.91^**^	3.92^**^	3.93^**^	3.95^**^
Individual-level controls	Age	0.05	0.03	0.03	0.05	0.03	0.04	0.05
	Sex	−0.02	−0.01	−0.01	−0.02	−0.01	−0.02	−0.02
	Education	0.08	0.06	0.05	0.11^*^	0.09	0.08	0.08
Team level controls	Team size	0.02	0.02	0.01	−0.02	−0.02	−0.02	0.01
Independent variable	TC		0.26^**^	0.24^**^		0.22^**^	0.08	
Mediator	PS						0.34^**^	0.25^**^
Individual-level moderator	KSSE							0.21^**^
Team level moderator	TV			0.07				
Individual-level interaction	PS × KSSE							0.28^**^
Cross-level interaction	TC × TV			0.19^**^				
*R*^2^ (individual level)		0.03	0.12^**^	0.20^**^	0.05^*^	0.15^**^	0.24^**^	0.36^**^
∆*R*^2^ (individual level)			0.09^**^	0.08^**^		0.10^**^	0.09^**^	0.12^**^

*N*_I_ = 282. N_T_ = 37. M represents Model; Sex: male = 1, female = 0; Education: high school or less = 1, bachelor’s degree = 2, master’s degree or higher = 3.

* *p* < 0.05;

** *p* < 0.01.

The three-step method (Baron and Kenny, 1986) was employed to test PS’s mediating effect. First, the results of H1 showed that TC positively affects KSB. Second, the results showed that TC is also positively related to PS (M2; *γ* = 0.22, *p* < 0.01). Third, when TC and PS were included into the equation of KSB, the effect of TC was less significant (M6; *γ* = 0.08, *ns*), whereas the effect of PS was significant (M6; *γ* = 0.34, *p* < 0.01). Thus, a fully mediating effect of PS was demonstrated based on these results. Moreover, to further confirm this mediating effect, we conducted a bias-corrected 95% confidence interval (CI) with 1,000 samples to assess the indirect effect. The results indicated that the indirect effect of TC on KSB through PS was significant (B = 0.12, Standard error [SE] = 0.03, CI [0.05, 0.21]). Thus, H2 was supported.

**Figure 2:**
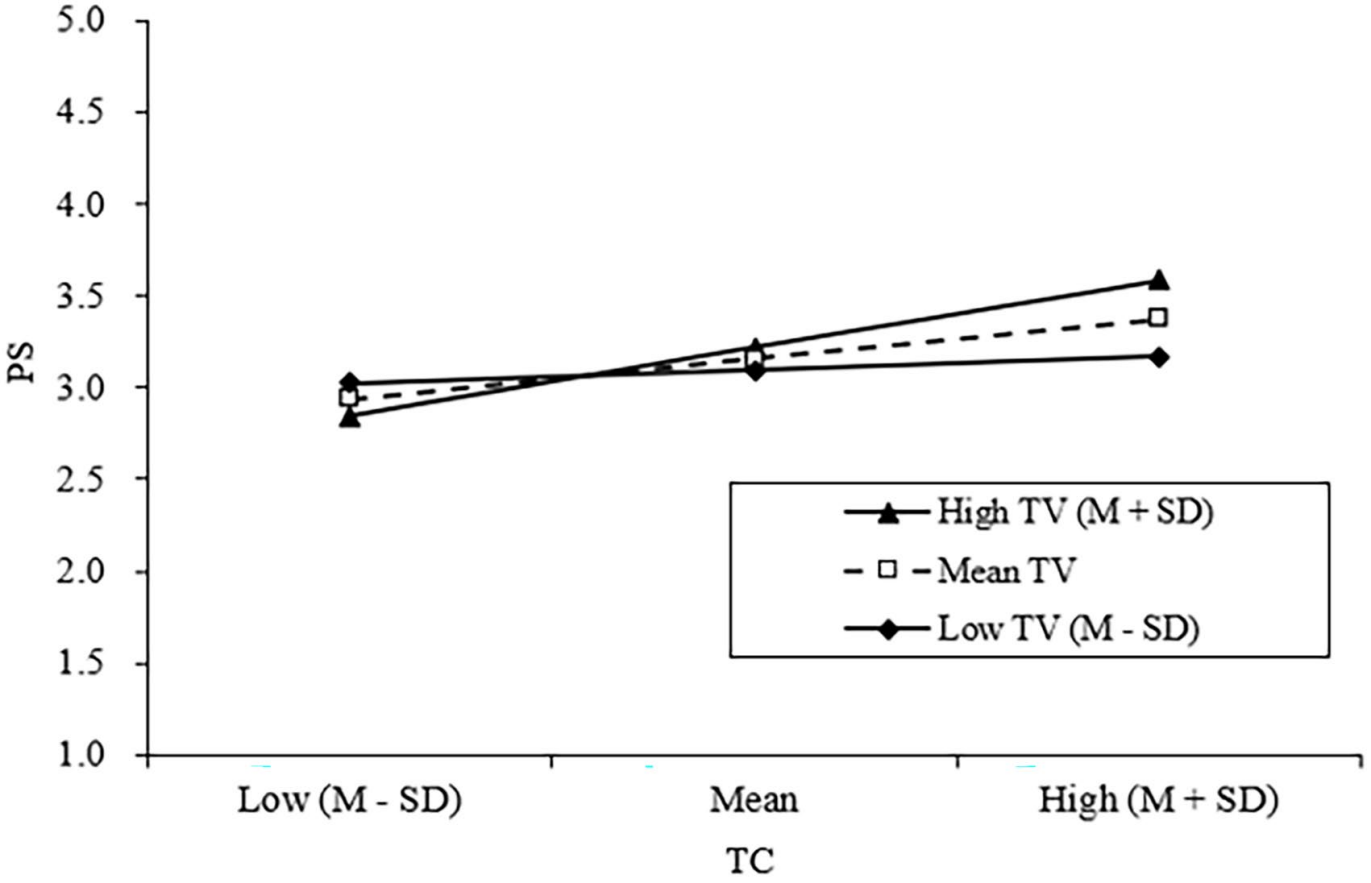
Interaction effect of TC and TV on PS.

**Figure 3:**
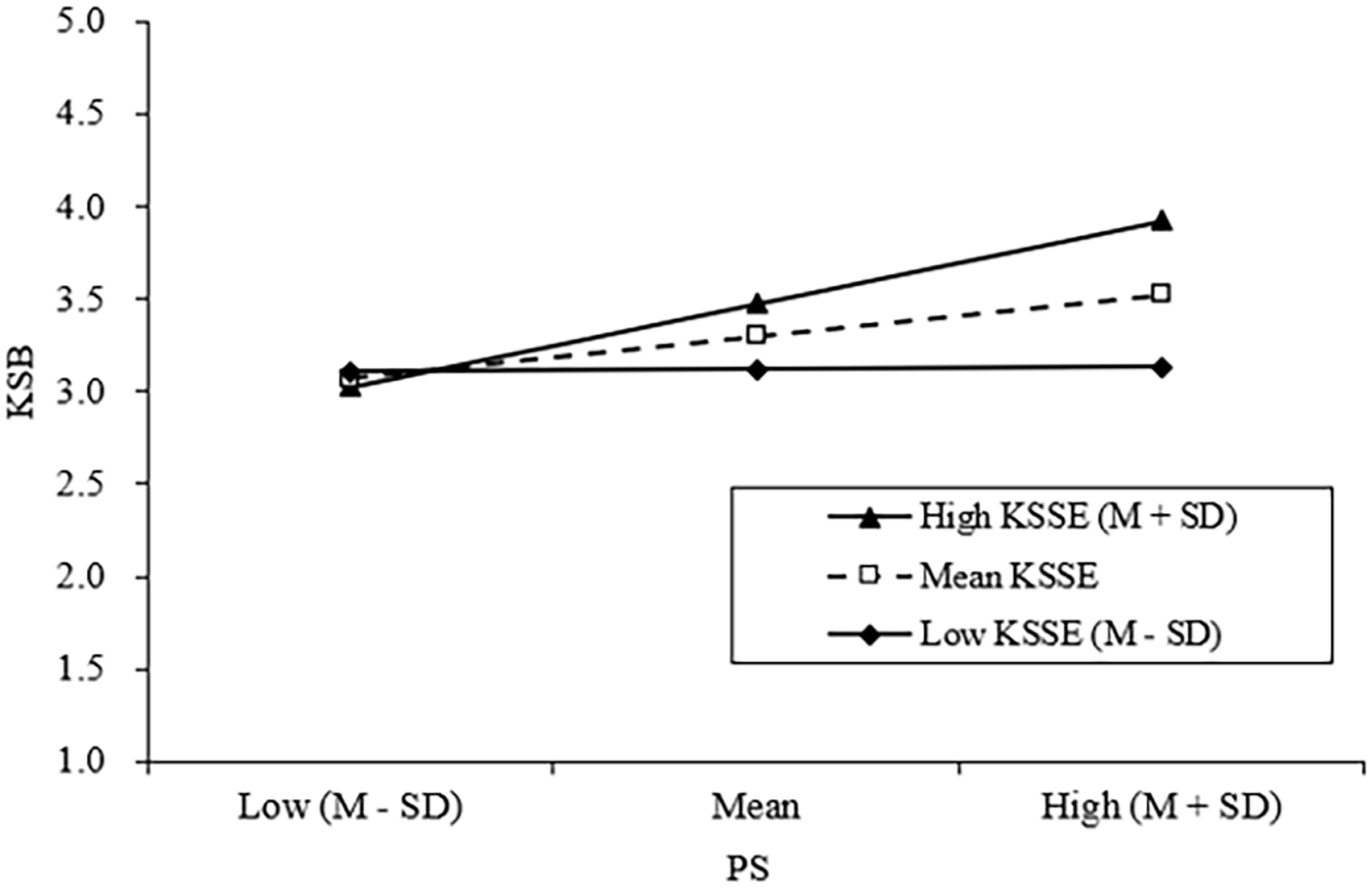
Interaction effect of PS and KSSE on KSB.

H3 and H5 proposed that TV and KSSE moderate the TC–PS and PS–KSB relationships at the team and the individual levels, respectively. The results in Table 3 showed that the effects of the cross-level interaction of TC and TV and the interaction of PS and KSSE were both significant (M3; *γ* = 0.19, *p* < 0.01; M7; *γ* = 0.28, p < 0.01), demonstrating ampliative effects of TV and KSSE. We further plotted these moderating effects (see Figures 2, 3) and computed the slopes at different TV and KSSE levels (see Table 4). Regarding the moderating effect of TV, results showed that when TV was high [mean + Standard Deviation (SD)], TC was significantly associated with PS (*B* = 0.37, *p* < 0.001). In contrast, when it was low (mean−SD), the relationship between TC and PS was less significant (*B* = 0.11, *p* < 0.05). Thus, H3 was supported. Regarding the moderating effect of KSSE, results showed that when KSSE was high (mean + SD), PS was significantly associated with KSB (*B* = 0.40, *p* < 0.001). In contrast, when it was low (mean−SD), PS would have a nonsignificant effect on KSB (*B* = 0.02, *ns*). Thus, H5 was supported.

**Table 4:** Summary of the simple slope tests.

Moderator levels	B	SE	*t*	*p*
Low TV	0.11	0.04	1.62	0.014
High TV	0.37	0.07	4.63	< 0.001
Low KSSE	0.02	0.01	0.03	0.813
High KSSE	0.40	0.08	5.76	< 0.001

Low refers to one SD below the mean; High refers to one SD above the mean; SE refers to standard error.

H4 and H6 predicted that TV and KSSE moderate the whole TC–PS–KSB relationship. To test these two hypotheses, we adopted Mplus 7.0 to examine the conditional indirect effects of TC on KSB through PS at different levels of TV and KSSE. We computed the normal distribution-base 95% CI for these effects. The results in Table 5 showed that when TV and KSSE were high (mean + SD), the indirect effects were significant (TV +1 SD, Estimate = 0.15, SE = 0.04, CI [0.06, 0.29]; KSSE +1 SD, Estimate = 0.27, SE = 0.07, CI [0.13, 0.46]), whereas when they were low (one SD below the mean), the indirect effects were insignificant (TV-1 SD, Estimate = 0.04, SE = 0.02, CI [−0.05, 0.08]; KSSE-1 SD, Estimate = 0.02, SE = 0.02, CI [−0.03, 0.04]). Thus, H4 and H6 were supported.

**Table 5:** Moderated mediation results for KSB across levels of TV and KSSE.

Moderator levels	Conditional indirect effect	SE	95% CI	
			Lower	Upper
Low TV	0.04	0.02	−0.05	0.08
High TV	0.15	0.04	0.06	0.29
Low KSSE	0.02	0.02	−0.03	0.04
High KSSE	0.27	0.07	0.13	0.46

Low refers to one SD below the mean; High refers to one SD above the mean; SE refers to standard error.

## Discussion and conclusion

### Key findings

This study aimed to uncover the complex nature of the linkage between TC and KSB in virtual teams by examining its psychological mechanism and the potential contingencies. We developed a multilevel moderated mediation model of TC and KSB where PS was employed as a mediator, whereas TV and KSSE were employed as team level and individual-level moderators, respectively. Consistent with our hypothesis, we established that TC positive correlates with KSB, and PS fully mediates this relationship. Furthermore, we found the following moderating effects: (a) TV acts as a team level moderator altering the way TC affects PS and the whole TC–PS–KSB effect. The relationship between TC and PS and the mediating effect of PS in the TC–KSB relationship is stronger in higher virtual environments. (b) KSSE exerts a moderating effect at the individual level, such that when KSSE is higher, the effect of PS on KSB and the mediating effect of PS are both stronger.

### Theoretical implications

Our study contributes to the existing literature in multiple ways. First, consistent with previous studies (e.g., Fang and Chiu, 2010; Zhang et al., 2010; Pangil and Chan, 2014), our findings reinforce that trust can effectively foster collaborative behaviors, such as KSB in virtual environments. However, our study is also distinctive from these studies and makes complementary contributions. Most prior work on the trust-KSB relationship considers trust as a general construct without clearly specifying different referents of it (e.g., Fang and Chiu, 2010). Although several studies have distinguished trust targets, most focus on trust in vertical referents, such as supervisors, upper management, and organizations, neglecting the other types of referents, specifically horizontal coworkers (Tan and Lim, 2009). Our study extends this line of research by highlighting the facilitating roles of TC on KSB. This research extension is of great value because the prevalence of deployed work teams such as virtual teams and the increasingly interdependent character of tasks underline the urgency and importance of examining the interaction qualities among coworkers. Moreover, to the best of our knowledge, our study is among the first to provide empirical evidence on the TC–KSB relationship in virtual teams.

Second, our study unveils a pivotal intervening mechanism in the TC–KSB relationship. Extant previous studies adopt social exchange theory as a theoretical perspective to demonstrate the relationship between trust and KSB (e.g., Chiu et al., 2006; Wu et al., 2009). Specifically, Chiu et al. (2006) stated that trust can maintain social exchange among employees thereby leading to good quality of KSB. Wu et al. (2009) argued that TC is considered an essential component in a social exchange relationship and higher levels of TC engenders better quality of social exchange relationship thereby facilitating more KSB. To understand how virtual team members process social information from trustworthy coworkers, the present study identifies PS as a motivational route which in turn affects KSB. Specifically, based on SIPT, the casual chain of why TC positively affects KSB is portrayed as follows: first, virtual team members within a collaborative social context, such as surrounding by trustworthy colleagues, are likely to process social cues from these colleagues in a way that generate relaxed, agreeable, and safe feelings, thereby leading to high levels of PS; second, these high PS employees will exhibit more KSB because they have few negative concerns about sharing actions. This specification contributes to the trust-KSB literature in two ways: first, it provides a credible description of the complex “black box” in this relationship and second, it demonstrates that trustworthy colleagues are a salient source of information which can be processed socially by virtual team members and then influence their psychological state and subsequent behaviors. In addition, the positive relationship between TC and PS echoes previous research that calls for clarifying the conceptual differences between trust and PS (Edmondson et al., 2004). For example, Edmondson et al. (2004) study argued that TC focused on others’ appropriate behaviors, whereas PS focused on individuals’ behaviors and consequences. Based on this argument, our findings showed that trust in other team members could create a favorable climate in virtual teams, reducing individuals’ negative concerns about their behaviors. Thus, our study distinguished these two “intrapsychic states” (i.e., trust and PS) from a conceptual perspective and connected these two similar constructs by empirically testing their relationship.

Third, our study established potential boundary conditions for the TC–PS–KSB relationship at different levels (i.e., team and individual). From a team perspective, the findings suggested that the direct relationship between TC and PS and the indirect relationship between TC and KSB through PS were conditional on TV. According to SIPT, employees need to process social information both from significant individuals (i.e., colleagues) and environmental characteristics (i.e., TV). More importantly, social cues from uncertain and ambiguous contexts, such as high TV environment, are likely to make other information, such as information from colleagues, more salient for employees to process (Lin et al., 2022). Specifically, in our study, greater degrees of TV were found to enhance the positive relationship between TC and PS and the mediating effect of PS in the TC-KSB relationship. In other words, as teams become more virtual, the positive roles of TC and PS become more prominent. This result concurs with a previous meta-analysis (Breuer et al., 2016), which showed that high TV contexts might increase team members’ perception of uncertainties, misunderstandings, conflicts, and risks at the workplace, thereby elevating their needs for trust and PS. However, our findings contradict Bierly et al. (2009) study that higher TV levels render trust less important for facilitating employees’ cooperation behaviors. A possible explanation for this contradiction, as Bierly et al. argued, is that a high TV environment might increase task independence and lead to less communication among team members. In contrast, we emphasized that virtual team members must work interdependently through electronical tools to achieve a common goal. In this regard, our study contributes to the virtual team literature by adding new empirical evidence for the inconsistent moderating effects of TV.

Regarding the individual-level moderators, we examined the moderating effect of KSSE in the TC–PS–KSB relationship based on social cognitive theory. Social cognitive theory suggests that an important barrier that hinders employees’ KSB in virtual environment is called self-efficacy deficits (Hsu et al., 2007). Consistent with this argument, our study revealed that KSSE could provide a “can do” attitude which effectively enhances the positive effects of PS on KSB and its mediating effect. Although previous studies have demonstrated positive relationship between PS and other work-related behaviors, such as voice, learning behaviors, KSB, and citizenship behaviors (Frazier et al., 2017), few have empirically examined the boundary conditions in these processes. Similarly, KSSE has usually been considered the main effect variable in predicting KSB and seldom examined its moderating effect (Hsu et al., 2007). Our study addresses these issues by empirically examining the combined effects of a psychological state (i.e., PS) and a cognitive self-evaluation (i.e., KSSE) on virtual team members’ KSB, which is a rewarding complement for the existing literature.

### Managerial implications

Our study also yields some valuable practical implications for virtual managers. First, our study showed that TC was a significant predictor for virtual team members’ KSB and PS, and its positive effects would increase as the team becomes more virtual. Thus, establishing trust and smooth communication among virtual team members is worth evaluating. The most important stage for building trust among team members is the team’s start-up (Pangil and Chan, 2014). Virtual team managers should grasp this critical stage and take some constructive actions to help establish TC. These actions could include organizing an informal face-to-face or a hybrid form of conversation, providing detailed personal resumes to team members, and encouraging team members to interact through social media. Beyond the start-up stage, sustaining the trust relationship is also a significant problem (Lin, 2007). Virtual teams should employ up-to-date electronic interactive mediums to increase the communication quality among team members (Pangil and Chan, 2014). In addition, proximal and shared experiences, such as hosting online dinners or online game contests, can help facilitate effective exchanges among team members, thus developing trust among them (Lin, 2007).

Second, our findings suggested that PS plays a critical mediating role in the TC–KSB relationship. Although TC can act as an antecedent of PS, leader relations also play important roles in elevating PS (Frazier et al., 2017). Thus, virtual team managers should help improve team members’ PS. Managers should use an open and truthful way to communicate with members and avoid unnecessary criticism (Zhang et al., 2010; Men et al., 2020), creating an atmosphere where voicing and sharing are safe.

Finally, the last managerial implication relates to the enhancement of KSSE. Our study confirmed that KSSE was an important amplifier for PS’ effects on KSB. Therefore, managers should conduct strategies to elevate virtual team members’ KSSE. For example, managers need to frequently provide positive and timely feedbacks to members’ sharing behaviors, such as appreciating a member’s sharing behavior itself, praising the member’s sharing ability, and acknowledging the quality of the shared knowledge. In addition, some online sharing training programs and support mechanisms should also be considered (Hao et al., 2019b).

### Limitations and future directions

Our study is not without limitations. First, the data of all variables were collected from the same source (i.e., virtual team members). Although we used Podsakoff et al.’s (2003) method to collect lagged data to minimize the impact of CMB, the problem cannot be completely eliminated. In addition, the social desirability bias with self-reported data is another noteworthy problem (Presser and Stinson, 1998), specifically for some favorable behaviors, such as KSB. Thus, future studies should collect data from different sources, such as leaders or colleagues, to validate the results. Second, the cross-sectional design prevents us from clearly explaining the causalities among the constructs. Experimental and longitudinal studies on this topic are extremely encouraged in the future. Third, our findings were based on the data of three internet companies. Extrapolating these findings to other industries requires extreme caution. Reproducing the study in other industries is highly recommended.

Fourth, we built a multilevel theoretical model and controlled several variables at the individual (i.e., age, sex, and education) and the team level, such as team size. However, some other variables may have effects on team members’ KSB. For example, a previous study showed that organizational culture was an important predictor for KSB (Serenko and Bontis, 2016). Future studies should consider such variables as controls to examine whether our model can be validated at the organizational level. Finally, other plausible constructs may alter how TC affects KSB through PS. For example, Men et al. (2020) showed that team climate, such as mastery climate, may influence the effects of PS on knowledge-relevant behaviors. Pinjani and Palvia (2013) argued that collaborative capabilities of available technology and task interdependence could moderate the relationship between trust and KSB in global virtual teams. Thus, in the future, more potential moderators at different levels should be examined in the TC–PS–KSB relationship.

### Conclusion

In this study, we extend our knowledge regarding the relationship between TC and KSB. Drawing on SIPT and social cognitive theory, we unfold the psychological mechanism (i.e., PS) in the TC–KSB relationship as well as recognizing boundary conditions (i.e., TV and KSSE) for these relationships. Specifically, our results demonstrate that TC foster PS, leading to high degree of KSB. Moreover, this process can be magnified by high TV and high KSSE. These findings make substantial contributions to the existing literature and provide some promising avenues for future studies.

## Data availability statement

The original contributions presented in the study are included in the article/supplementary material; further inquiries can be directed to the corresponding authors.

## Ethics statement

Ethical review and approval were not required for the study on human participants in accordance with the local legislation and institutional requirements. Written informed consent for participation was not required for this study in accordance with the national legislation and the institutional requirements.

## Author contributions

All authors listed have made a substantial, direct, and intellectual contribution to the work and approved it for publication.

## Funding

This work was supported by the Fundamental Research Funds for the Central Universities and the Research Funds of Renmin University of China (No. 21XNF043; No. 21XNL019; No. 22XNH004). The content is solely the responsibility of the authors. The funding source had no role in study design, data collection, analysis and interpretation, the writing of the manuscript or the decision to submit the paper for publication.

## Conflict of interest

The authors declare that the research was conducted in the absence of any commercial or financial relationships that could be construed as a potential conflict of interest.

## Publisher’s note

All claims expressed in this article are solely those of the authors and do not necessarily represent those of their affiliated organizations, or those of the publisher, the editors and the reviewers. Any product that may be evaluated in this article, or claim that may be made by its manufacturer, is not guaranteed or endorsed by the publisher.

## Appendix A

Measures of constructs.

**Table:**

Construct	Source
**Trust in coworkers (TC)**	Lin (2007)
TC1. I consider my coworkers as people who(m) can be trusted.	
TC2. I consider my coworkers as people who(m) can be counted on to do what is right.	
TC3. I consider my coworkers as people who(m) can be counted on to get the job done right.	
TC4. I consider my coworkers as people who(m) are always faithful.	
TC5. I consider my coworkers as people who(m) I have great confidence in.	
**Psychological safety (PS)**	Liang et al. (2012)
PS1. In my work unit, I can express my true feelings regarding my job.	
PS2. In my work unit, I can freely express my thoughts.	
PS3. In my work unit, expressing your true feelings is welcomed.	
PS4. Nobody in my unit will pick on me even if I have different opinions.	
PS5. I’m worried that expressing true thoughts in my workplace would do harm to myself (reverse-coded).	
**Team virtuality (TV)**	Chudoba et al. (2005)
Team distribution	
TV1. I collaborate with team members in different time zones.	
TV2. I work with team members *via* internet-based conferencing applications.	
TV3. I collaborate with team members that I have never met face-to-face.	
TV4. I collaborate with team members who speak different native languages or dialects than your own.	
Workplace mobility	
TV5. I work at different departments.	
TV6. I have professional interactions with people outside the team.	
TV7. I work with mobile devices.	
TV8. I work at home during normal business days.	
TV9. I work while traveling, for example, at airports or hotels.	
Variety of practices	
TV10. I work on projects that have changing team members.	
TV11. I work with teams that have different ways to track their work.	
TV12. I work with people that use different collaboration technologies.	
**Knowledge sharing self-efficacy (KSSE)**	Lin et al. (2009)
KSSE1. I have confidence in my ability to provide knowledge that other members in this virtual community consider valuable.	
KSSE2. I have the expertise, experiences, and insights needed to provide knowledge that is valuable for other members in this virtual community.	
KSSE3. I have confidence in responding or adding comments to messages or articles posted by other members in this virtual community.	
**Knowledge sharing behavior (KSB)**	Lin et al. (2009)
KSB1. I frequently participate in knowledge-sharing activities and share my knowledge with others in this virtual community.	
KSB2. I usually spend a lot of time conducting knowledge-sharing activities in this virtual community.	
KSB3. When discussing a complicated issue, I am usually involved in the subsequent interactions.	
